# Dietary patterns reflecting healthy food choices are associated with lower serum LPS activity

**DOI:** 10.1038/s41598-017-06885-7

**Published:** 2017-07-26

**Authors:** Aila J Ahola, Mariann I. Lassenius, Carol Forsblom, Valma Harjutsalo, Markku Lehto, Per-Henrik Groop

**Affiliations:** 1 Folkhälsan Institute of Genetics, Folkhälsan Research Center, Helsinki, Finland; 2 Abdominal Center Nephrology, University of Helsinki and Helsinki University Central Hospital, Helsinki, Finland; 3 Research Programs Unit, Diabetes and Obesity, University of Helsinki, Helsinki, Finland; 4 National Institute for Health and Welfare, Diabetes Prevention Unit, Helsinki, Finland; 5 Baker IDI Heart and Diabetes Institute, Melbourne, Australia

## Abstract

Gram-negative bacteria-derived lipopolysaccharides (LPS) are associated with various negative health effects. Whether diet is associated with LPS, is an understudied phenomenon. We investigated the association between diet and serum LPS activity in 668 individuals with type 1 diabetes in the FinnDiane Study. Serum LPS activity was determined using the Limulus Amoebocyte Lysate assay. Diet was assessed with a food frequency questionnaire (FFQ) section of a diet questionnaire and a food record. The food record was used to calculate energy, macronutrient, and fibre intake. In a multivariable model, energy, macronutrient, or fibre intake was not associated with the LPS activity. Using factor analysis, we identified seven dietary patterns from the FFQ data (“Sweet”, “Cheese”, “Fish”, “Healthy snack”, “Vegetable”, “Traditional”, and “Modern”). In a multivariable model, higher factor scores of the Fish, Healthy snack, and Modern patterns predicted lower LPS activity. The validity of the diet questionnaire was also investigated. The questionnaire showed reasonable relative validity against a 6-day food record. The two methods classified participants into the dietary patterns better than expected by chance. In conclusion, healthy dietary choices, such as consumption of fish, fresh vegetables, and fruits and berries may be associated with positive health outcomes by reducing systemic endotoxaemia.

## Introduction

Lipopolysaccharides (LPS), also frequently called endotoxins, are lipid-soluble outer-membrane components of Gram-negative bacteria^1^. Among these bacteria are many pathogens, but also much of the commensal population of the human gut (i.e. *Bacteroides*). Indeed, the bacteria colonized in the intestinal tract are a major source of LPS in humans^2^. Importantly, LPS may be translocated from the intestine into the systemic circulation. During the prandial state, for example, the lipid regions of the LPS particle are incorporated into the forming chylomicrons, thereby enabling its absorption^3^. The paracellular translocation of LPS, on the other hand, may be enhanced upon impairment of the integrity of the mucosal epithelial barrier.

Binding of LPS-protein complexes to the toll-like receptor 4 activates cellular NF-κB signalling pathway which, in turn, leads to production of various proinflammatory cytokines and chemokines^4^. Elevated serum LPS activity levels have been linked with a number of unfavourable changes in human health. In diabetes and in obese subjects, high LPS levels have shown to be negatively correlated with insulin sensitivity^5^. Accordingly, LPS has been associated with obesity and type 2 diabetes^6, 7^, as well as progression of non-alcoholic fatty liver disease^8^. The LPS-induced secretion of proinflammatory cytokines may also lead to endothelial dysfunction, plaque formation and rupture, oxidation of LDL-cholesterol particles, and thrombogenesis, thus contributing to the development of cardiovascular disease^6, 9^. In type 1 diabetes, we have previously shown that serum LPS activity is associated with the metabolic syndrome^10^, visceral fat mass^11^, and the development of diabetic nephropathy^12^.

Despite the obvious importance of LPS in human health, only relatively few studies have been conducted to assess whether LPS levels could be affected by dietary means. In one of these studies, Erridge *et al*. reported increase in circulating endotoxin levels, in healthy subjects, following a high-fat meal^13^. Similarly, cream consumption induced a rise in the plasma LPS concentrations, in healthy normal-weight adults, while isoenergetic intake of glucose had no such effect^14^. In contrast to these observations, however, a high-fat low-carbohydrate ketogenic diet exhibited lower proinflammatory cytokine levels in an LPS-induced fever model^15^. *In vitro*, fruit and vegetable extracts have shown the capacity to inhibit LPS-stimulated inflammatory responses^16^. Finally, orange juice consumption with a high-fat, high-carbohydrate meal prevented meal-induced increase in endotoxin concentration^17^.

Amongst the available studies, we were able to identify only one focusing on the association between dietary patterns and LPS levels^18^. In that study, a 6-month intervention with either a standard Healthy Eating diet or a modified Mediterranean diet was, insufficient to show any changes in the LPS-binding protein concentrations^18^.

In this paper we report the results of an observational study investigating the association between diet and serum LPS activity in a group of well-characterized individuals with type 1 diabetes. An approach was taken to study the dietary intake both at the level of macronutrient intake (food record data) and at the level of dietary patterns (food frequency questionnaire section of a diet questionnaire, FFQ). We hypothesized that a healthy diet would be associated with lower endotoxin levels. Moreover, we also report the results of a validation study, where the relative validity of the diet questionnaire against a 6-day food record was examined in a large population of individuals with type 1 diabetes.

## Results

### Food frequency questionnaire data

A total of 668 individuals with type 1 diabetes completed the FFQ and fulfilled the inclusion criteria (Table 1). Of these participants, 47% were men, and the mean ± SD age was 44 ± 13 years. The median (interquartile range) serum LPS activity of the population was 0.37 (0.30–0.49) EU/ml.

**Table 1:** Basic characteristics of the study population.

	FFQ data n = 668	Food record data n = 542	*P*
Men, %	47	44	0.417
Age, years	44 ± 13	45 ± 13	0.346
LPS, EU/ml	0.37 (0.30–0.49)	0.37 (0.30–0.49)	0.693
Diabetic nephropathy, %	18	16	0.347
Hard cardiovascular event, %	14	13	0.668
Insulin dose, IU/kg	0.62 (0.48–0.78)	0.61 (0.48–0.77)	0.568
BMI, kg/m^2^	25 (23–28)	26 (23–28)	0.737
Systolic blood pressure, mmHg	136 (124–149)	135 (124–148)	0.684
Diastolic blood pressure, mmHg	77 (71–84)	76 (71–84)	0.465
HbA_1c_, %	8.0 (7.4–8.7)	8.0 (7.3–8.7)	0.614
HbA_1c_, mmol/mol	63.9 (57.4–71.6)	63.9 (56.3–71.6)	0.614
Total cholesterol, mmol/l	4.5 (4.0–5.1)	4.5 (4.0–5.1)	0.870
HDL cholesterol, mmol/l	1.52 (1.28–1.85)	1.53 (1.30–1.86)	0.624
Triglyceride, mmol/l	0.95 (0.72–1.34)	0.95 (0.72–1.32)	0.680
Current smoking, %	13	12	0.659
Lipid lowering medication, %	34	34	1.000
Antihypertensive medication, %	47	46	0.770
Physical activity at least once a week, %	80	82	0.269

In all 668 participants completed the food frequency questionnaire (FFQ). Of these, 542 additionally returned the food record. Data are presented as frequency (%), mean ± standard deviation, or median (interquartile range). LPS, lipopolysaccharide; Hard cardiovascular event, any major cardiovascular event (acute myocardial infarction, coronary bypass, stroke, amputation, peripheral vascular disease).

Seven dietary factors (or patterns) with high degree of inter-correlation were formed from the FFQ (Table 2). Based on the contents of these clusters, they were named “Sweet”, “Cheese”, “Fish”, “Healthy snack”, “Vegetable”, “Traditional”, and “Modern”. The factor score of the Sweet -diet pattern correlated negatively with the LPS activity (*r = *−0.080, *P = *0.039). In the model only adjusted for all the other dietary patterns, higher factor scores of the Fish and the Healthy snack patterns were associated with lower serum LPS activity (Table 3, Model 1). After further adjusting for sample storage time, age, sex, smoking, physical activity and diabetic nephropathy status, higher factor scores of the Fish [B (95% Confidence Interval), *P*-value; −0.020 (−0.035 – −0.006), 0.007], the Healthy snack [−0.023 (−0.043 – −0.002), 0.029], and the Modern [−0.027 (−0.050 – −0.004), 0.020] diet patterns significantly predicted lower serum LPS activity (Table 3, Model 3).

**Table 2:** Factor analysis-derived dietary patterns (n = 668).

Dietary pattern	Eigenvalue	% of variance	Included food items	Factor loadings
Sweet	2.09	11.0	Sweets and chocolate	0.661
			Sweet pastry	0.579
			Ice cream	0.330
			Yoghurt	0.245
Cheese	1.87	9.9	High-fat cheese	0.975
			Low-fat cheese	−0.260
Fish	1.61	8.5	Fish dishes	0.985
Healthy snack	1.40	7.4	Fruits and berries	0.637
			Fresh vegetables	0.473
			Yoghurt	0.411
			Low-fat cheese	0.285
			Soft drinks	−0.240
Vegetable	1.35	7.1	Cooked vegetables	0.717
			Legumes	0.533
			Fresh vegetables	0.291
Traditional	1.19	6.3	Meat dishes	0.701
			Potatoes	0.476
			Sausages and cold cuts	0.347
Modern	1.04	5.4	Poultry	0.487
			Pasta and rice	0.439
			Meat dishes	0.436
			Fried and grilled foods	0.283
			Fresh vegetables	0.202

Eigenvalues are the variances of the factors; % of variance shows the per cent of total variance accounted by each factor; the factor loadings represent the correlation of each food item with the given dietary pattern. Factors are formed from the food frequency questionnaire data.

**Table 3:** Associations between factor analysis-derived dietary patterns and serum LPS activity in 668 individuals.

	B	95% Wald CI	*P*
Model 1			
Sweet	−0.016	−0.033–0.002	0.082
Cheese	−0.003	−0.017–0.011	0.714
Fish	−0.020	−0.034 – −0.006	0.005
Healthy snack	−0.020	−0.038 – −0.001	0.038
Vegetable	−0.007	−0.025–0.011	0.419
Traditional	0.005	−0.013–0.023	0.587
Modern	−0.012	−0.031–0.008	0.255
Model 2			
Sweet	−0.017	−0.034–0.000	0.052
Cheese	−0.008	−0.022–0.005	0.225
Fish	−0.020	−0.034 – −0.006	0.005
Healthy snack	−0.013	−0.032–0.006	0.178
Vegetable	−0.005	−0.022–0.013	0.587
Traditional	0.010	−0.008–0.028	0.271
Modern	−0.023	−0.044 – −0.003	0.024
Storage time	−0.018	−0.023 – −0.012	0.001
Age	−0.002	−0.003 – −0.001	0.003
Sex	0.003	−0.027–0.032	0.861
Model 3			
Sweet	−0.008	−0.026–0.010	0.387
Cheese	−0.006	−0.021–0.009	0.396
Fish	−0.020	−0.035 – −0.006	0.007
Healthy snack	−0.023	−0.043 – −0.002	0.029
Vegetable	−0.004	−0.024–0.015	0.652
Traditional	0.006	−0.013–0.026	0.519
Modern	−0.027	−0.050 – −0.004	0.020
Storage time	−0.018	−0.023 – −0.012	0.001
Age	−0.002	−0.003 – −0.001	0.003
Sex	0.000	−0.031–0.031	0.991
Smoke	−0.029	−0.071–0.014	0.187
Physical activity	0.019	−0.018–0.056	0.304
Diabetic nephropathy	0.000	−0.039–0.039	0.993

Generalized linear model. Factors are formed from the food frequency questionnaire data.

### Food record data

A total of 542 individuals (44% men, age 45 ± 13 years) completed the food record (Table 1). The median (interquartile range) energy [7694 (6468–9194) kJ)], carbohydrate [44.0 (39.3–48.1) E%)], fat [35.2 (30.9–38.9) E%)], protein [16.6 (14.8–18.5) E%)], and alcohol [1.1 (0–3.6) E%)] intake, of the population, was calculated. Of these dietary variables, carbohydrate intake correlated negatively with the serum LPS activity (*r = *−0.108, *P = *0.012). Two models were constructed to study the association between macronutrient intake and serum LPS activity (Table 4). In the first model, dietary variables were entered as percentages of total energy intake, and in the second model as grams. Both models were additionally adjusted for energy and fibre intake, sample storage time, age, sex, smoking, physical activity, and diabetic nephropathy status. In these models, none of the dietary variables predicted serum LPS activity levels.

**Table 4:** Associations between dietary intake (energy, macronutrient, and fibre intake) and serum LPS activity in 542 individuals.

	B	95% Wald CI	*P*
Model 1			
Energy, kJ	0.000	−0.001–0.001	0.925
Carbohydrates, E%	0.007	−0.008–0.022	0.366
Fats, E%	0.005	−0.009–0.020	0.472
Protein, E%	0.010	−0.006–0.027	0.215
Alcohol, E%	0.006	−0.010–0.022	0.445
Fibre, g	−0.001	−0.003–0.002	0.640
Model 2			
Energy, kJ	0.000	−0.001–0.001	0.525
Carbohydrates, g	0.001	−0.002–0.004	0.526
Fats, g	0.002	−0.005–0.009	0.642
Protein, g	0.002	−0.002–0.005	0.300
Alcohol, g	0.002	−0.004–0.007	0.604
Fibre, g	−0.001	−0.003–0.002	0.585

kJ, kilojoule; E%, percentage of total energy intake. Data on energy and nutrient intakes have been obtained from the food records. Both models have been adjusted for sample storage time, age, sex, smoking, physical activity, and diabetic nephropathy status. Generalized linear model.

We then studied the association between LPS activity and macronutrient intake using the multivariable nutrient density substitution model (Table 5). In these analyses, neither the isoenergetic substitutions between macronutrient intakes, nor the substitutions between fatty acid intakes were associated with the LPS activity.

**Table 5:** Association between LPS activity and dietary macronutrient intake (substitution model) in 542 individuals.

	B	95% Wald Confidence Interval	*P*
Macronutrients			
Carbohydrate (Fat)	0.006	−0.007–0.019	0.391
Carbohydrate (Protein)	−0.008	−0.035–0.018	0.535
Carbohydrate (Alcohol)	0.005	−0.018–0.027	0.689
Protein (Fat)	0.021	−0.010–0.053	0.185
Protein (Alcohol)	0.019	−0.014–0.053	0.254
Fat (Alcohol)	0.000	−0.023–0.023	0.976
Fatty acids			
Polyunsaturated fatty acids (Saturated fatty acids)	−0.020	−0.088–0.048	0.564
Polyunsaturated fatty acids (Monounsaturated fatty acids)	0.011	−0.087–0.108	0.830
Monounsaturated fatty acids (Saturated fatty acids)	0.014	−0.068–0.095	0.743

In each model, all but one macronutrient (the one in the parenthesis) is included together with energy intake and selected covariates (sample storage time, age, sex, smoking, physical activity, and diabetic nephropathy status). The models with fatty acids, are additionally corrected for trans fatty acid intake. The B represents an increase or decrease in the LPS activity when, in an isoenergetic condition, the intake of a given macronutrient or fatty acid is increased by 5 per cent of the total energy intake, at the expense of the macronutrient or fatty acid in the parenthesis. Thus in the first row, for example, the B shows the (non-significant) increase in the LPS activity when carbohydrate intake is increased by 5 per cent of the total energy intake at the expense of fat intake, while keeping the energy intake constant. Data on energy and nutrient intakes have been obtained from the food records. Generalized linear model.

### Validation study

The basic characteristics of the study participants included in the validation study are shown in the Supplementary Table 1. The mean amounts of coffee, tea, milk, sour milk, yoghurt or curd, and bread, reported in the diet questionnaire and in the 6-day food record, were significantly correlated (*P < *0.001, all) (Supplementary Table 2). Also the proportions of individuals reporting the consumption of filtered coffee, fat free milk, fat free sour milk, low fat yoghurt or curd, rye bread, and vegetable based spread were similar between the two methods (*P < *0.001, all).

The mean consumption frequencies of the food groups reported in the FFQ part of the diet questionnaire and the 6-day food record are presented in Supplementary Table 3. The consumption frequencies of all the food groups were significantly correlated between the two methods (*P < *0.001, all).

A total of six reasonably well matching dietary patterns were produced in the factor analysis from the FFQ and the 6-day food record (Supplementary Table 4). As the food items in the Cheese pattern (high-fat cheese and low-fat cheese) were observed to load in the opposite directions, in the two analyses, we applied inverse scoring for the food record-derived factor scores when classifying participants into the quartiles. For all the dietary patterns, the cross-classification to the same quartile, as well as to the same or adjacent quartile, was higher than expected by chance (Supplementary Table 5). Also the proportion of individuals misclassified to the opposite quartile was lower than expected by chance in all dietary patterns. Moreover, all factor scores of the six dietary patterns were observed to correlate significantly (Supplementary Table 4) (P < 0.001, all).

Finally, correlations of energy and macronutrient intakes between the two separate 3-day food records were investigated, and all variables were observed to correlate significantly (*P* < 0.001). Highest correlation was observed for the reported energy intake (*r* = 0.73), and lowest for the polyunsaturated fat intake (*r = *0.43) (data not shown).

## Discussion

The potential role of bacterial endotoxins as proinflammatory mediators in the development of cardiovascular disease is increasingly recognized^19^. Notably, gram-negative bacteria, the source of LPS, are known to colonize the gastrointestinal tract of even apparently healthy individuals^20^. Indeed, the human gut is a major reservoir of LPS and, for these endotoxins, an important site of entry to the systemic circulation. Given that diet has the potential to modulate, not only the composition of the gut microbiota and its metabolic activity^21^, but also the integrity of the intestinal epithelium^22^, we hypothesized that dietary intake is associated with the level of endotoxaemia. In a population of individuals with type 1 diabetes, we observed that dietary patterns, reflecting food choices that are generally considered healthy, were associated with lower serum LPS activity. Amongst some of the food items, included in these dietary patterns, were fish, fresh vegetables, fruits and berries, yoghurt, pasta and rice, and poultry. Instead, no association was observed between dietary intake, measured at the level of macronutrients or fatty acids, and LPS.

Of the macronutrients, the role of dietary fats has gained particular interest in many of the previous LPS studies. Amongst other mechanisms, fat intake may induce endotoxaemia via enhanced intestinal absorption related to chylomicron formation^3^. High fat intake has also been shown to favour the numbers of LPS-containing Gram-negative bacteria in the gut at the expense of Gram-positive bacteria^23^. In human studies, an increase in postprandial endotoxaemia has been observed after a single high-fat meal^14, 24^. It could be speculated, however, that in the short-term feeding studies the amounts or proportions of fat consumed may be significantly higher than what is typically consumed as part of a regular diet. Other macronutrients, fibre, antioxidants and phytochemicals, present in the diet as a whole, may contribute to the complex associations that take place between the diet, gut microbes, and endotoxaemia. Finally, it has been suggested that total energy intake, rather than fat intake *per se*, confounds the previous observations related to dietary fat and endotoxaemia^25^.

Beyond studies focusing on specific macronutrient intake, only relatively few studies have been conducted on overall diet and endotoxaemia. In one such study, over a 6-month intervention with a standard Healthy Eating diet or a modified Mediterranean diet, no change in the plasma LPS-binding protein concentrations was observed, despite substantial increase in the intakes of fruits and vegetables and simultaneous reduction in fat intake^18^. The authors suspected that such interventions may show improvements in the LPS-binding protein levels only when instituted in populations with initially poor quality diets. In another study, a two-week intervention with 12 servings of fruits and vegetables per day, compared to period of 2 daily servings, exhibited a significantly lower *ex vivo* production of interleukin-6 in LPS-activated mononuclear cells^26^. As the two intervention diets were matched with respect to energy, macronutrient and fibre intake, the authors concluded that the effect was due to the difference in the phytochemical composition of the diets. In support of this hypothesis, isoflavone^27^ and quercetin^28^ consumption have been shown to produce beneficial responses in LPS-evoked endotoxaemia.

The current observations suggest the importance of an overall healthy diet in tackling the endotoxaemia. Many constituents of a healthy diet may contribute to the observed beneficial effects. For example, abundant intake of fibre may reduce LPS exposure not only via its ability to modify the microbial composition, but also through the production of short-chain fatty acids. Indeed, these end-products of microbial fermentation have shown to reduce the gut permeability by facilitating tight junction assembly^29^. Also, microbially produced butyrate stimulates the expression of intestinal alkaline phosphatase, which is involved in the regulation of chylomicron transport and detoxification of bacterial endotoxins^30^. Notably, in patients with type 2 diabetes, fibre supplementation exhibited a significant reduction in the LPS concentrations compared to the control group^31^. Consumption of fish, as part of a healthy diet may attenuate the disruption of the intestinal barrier function, as seen in studies of LPS-induced inflammation^32, 33^. Finally, potentially through the reduction in luminal cytotoxicity, lactobacilli growth and reduction of intestinal mucosal damage, dietary calcium appears to positively influence the composition of the gut microbiota and the intestinal barrier integrity^34^.

While our observations provide important insights into the association between dietary intake and endotoxaemia, the current study has also limitations that should be acknowledged. Importantly, the cross-sectional study design cannot give a definite answer to the question whether the diet affects LPS levels. Instead, our observations offer hypotheses that should be tested in intervention trials. The methods to study dietary intake have their unique limitations, as previously discussed^35^. Shortly, for a number of reasons, individuals may over- or under-report their dietary intake. Generally, such misreporting is thought to occur for food items perceived healthy and unhealthy, respectively. Should this have taken place, in the current study, it has most likely diluted the observations. Although the number of included participants was relatively large and the patients were well-characterised, some selection-bias may have taken place, which could limit the generalizability of the results. Indeed, compared to individuals volunteering to participate in a study, those declining may be less healthy or less health-conscious. Such selection-bias may have, for example, excluded proportionally higher number of individuals with diets perceived less optimal for health which, again, has a potential to dilute our observations. Finally, we cannot exclude that some residual confounding could explain our observations. One important confounder, not taken into account in the current study, is the potential impairment of the integrity of the gut. Some dietary constituents, such as gliadin^36^, has the potential to increase gut permeability which, again, could be associated with increased LPS absorption. Due to a small number of individuals adhering to a gluten-free diet, we were not able to address this issue in the current analyses.

Amongst the strengths of this study is that we measured dietary intake using both a macronutrient and a dietary pattern approach. With this dual approach, we were able to identify that diet, at the level of food items, is associated with serum LPS activity. However, dietary intake measured at the macronutrient level had no such association. Moreover, we investigated the role of macronutrient intake using two separate methods. First, all macronutrients were entered in a multivariable model in order to study whether their intake, as reported in the food records, was associated with the LPS activity. Next, we used the multivariable nutrient density substitution model, with a different rationale compared to the previous analysis. The idea behind the substitution model is that, by making different food-level choices, we can decrease (or increase) the intake of a particular macronutrient. In order to satisfy the energy requirement, however, one has to simultaneously increase (or decrease) the intake of another macronutrient. Indeed, the multivariable nutrient density substitution model takes into account this shift in the relative distribution of dietary macronutrients. Thus, we were able to calculate whether a five per cent increase in the consumption of one of the macronutrients, accompanied by a five per cent decrease in another macronutrient, had any effect on the level of LPS activity. While this is an appealing approach, occasionally used in the nutrition research^37, 38^, it has to be acknowledged that it is a mathematical model, and does not represent a real-life condition.

Finally the relative validity of the diet questionnaire, and subsequently the FFQ -section of the questionnaire, was investigated. The obtained results revealed that all the dietary variables investigated with the diet questionnaire and the 6-day food record were significantly correlated, and the questionnaire was able to correctly classify individuals in the formed dietary patterns. Moreover, data collected by the two separate 3-day food records, with a 2–3 month interval, showed significant correlation, suggesting that the no major changes in energy and macronutrient intakes take place in this population over this relatively short period.

In conclusion, our observations support the view that a healthy diet, as a whole, is associated with lower levels of endotoxaemia in type 1 diabetes. Instead, the habitual intake of energy, fibre, individual macronutrients or fatty acids has no relation to the serum LPS activity. The role of specific dietary patterns, in modifying endotoxaemia, should be investigated in an interventional setting. Moreover, whether dietary intake may, via its association with serum LPS levels, reduce the risk of diabetic complications, should be prospectively assessed.

## Methods

The study subjects were participants in the Finnish Diabetic Nephropathy (FinnDiane) Study (www.finndiane.fi). Since 1997, data from more than 5000 individuals have been collected. However, the collection of dietary data did not start until 2007. Therefore dietary data are available only from a subset of the FinnDiane Study population. Included in the current LPS-diet study were all participants in the FinnDiane Study with serum LPS activity measurements who had completed the diet questionnaire within two years from the study visit. The study protocol was approved by the Ethics Committee of Helsinki University Central Hospital, and the study was performed in accordance with the relevant guidelines and regulations. All subjects provided written informed consent prior to study participation.

During the regular visits to the attending physician, participants’ weight and height were measured, and body mass index (BMI, kg/m^2^) was calculated. Following a 10-minute rest, blood pressure was measured while seated. The second measurement followed after a 2-minute interval, and a mean of these measurements was calculated. Fasting (or following a light breakfast) blood samples were drawn and HbA_1c_ was determined locally using standardized assays. Serum lipid and lipoprotein concentrations were measured as previously described^39^. Serum LPS activity was measured with the Limulus Amebocyte Lysate assay from 1:5 diluted serum samples at 405 nm as an end-point assay (LAL, Hycult Biotechnology, the Netherlands). Data on smoking and insulin dose were self-reported. The reported insulin dose was divided by body weight to obtain daily insulin dose in IU/kg. The attending physician recorded data on patient’s medication and diabetes complications on a standardized form. Data on physical activity were collected as previously described^40^.

Urinary albumin excretion rate (AER) in at least two out of three timed 24-hour or overnight urine collections was used to assess the participants’ renal status. The following classifications were made: normal albumin excretion rate (AER < 20 µg/min or <30 mg/24 h), microalbuminuria (AER ≥ 20 and <200 µg/min or ≥30 and <300 mg/24 h), macroalbuminuria (AER ≥ 200 µg/min or ≥300 mg/24 h), and end-stage renal disease (ESRD) (in dialysis or with kidney transplant). Diabetic nephropathy was defined as macroalbuminuria or ESRD.

### Diet questionnaire

Dietary intake was measured using two separate methods, as previously described^41^. First, at the study visit, participants were provided a self-reported diet questionnaire to be completed. The food items included in the diet questionnaire and the types of data collected with the form are presented in Supplementary Table 6. The questionnaire also included a FFQ, where the consumption frequencies of a number of basic food items were queried (fish dishes, meat dishes, poultry, sausages and cold cuts, eggs, legumes, fresh vegetables, cooked vegetables, potatoes, pasta and rice, fruits and berries, high-fat cheese, low-fat cheese, yoghurt, ice cream, soft drinks, sweet pastries, sweets and chocolate, and fried and grilled foods). In this section, seven frequency response options were provided: several times per day, once a day, 4–6 times per week, 2–3 times per week, once a week, 1–3 times per month, and less frequently or never. In the current LPS-diet study, only data from the FFQ-section of the diet questionnaire are used. Here, the FFQ-derived data were used to form dietary patterns, representing the dietary intake at the level of food items, as described in the Statistical analyses in more detail. The factor scores of these dietary patterns were subsequently entered to the multivariable models to study the association between dietary patterns and serum LPS activity.

### Food record

Upon returning the diet questionnaire, participants were sent a 3-day exercise and food record to be completed. In this record, participants reported all foods and drinks consumed during the three allocated consecutive days (two weekdays and one weekend day). The 3-day record-keeping was repeated after 2–3 months in order to take some seasonal variation into account. AivoDiet software (version 2.0.0.1, AIVO, Turku, Finland), based on the Finnish National Food Composition Database, was used to calculate the mean daily energy, macronutrient (both as grams and as percentages of total energy intake, E%), and fibre intakes from the records. The intake of trans fatty acids was estimated by subtracting saturated, monounsaturated, and polyunsaturated fatty acid intakes from the total fat intake. Subsequently, trans fatty acid intake was only used to adjust the fatty acid models, and not as an independent variable. Data collected with the food records were used in the multivariable models to study the association between dietary intake, at the level of energy and macronutrient intake, and serum LPS activity. In the current LPS-diet analyses, data from individuals who completed the record for a minimum of three days were included.

### Validation study

For the validation study, we included data from all participants in the FinnDiane Study who had completed both the diet questionnaire and the 6-day food record (n = 1171). From the food record entries, data on all food items for which an unambiguous match was found in the diet questionnaire were extracted for subsequent analyses. For example, the numbers of cups of coffee, reported in the food record, were calculated and divided by the number of days the record was kept (six) to obtain the average of daily cups of coffee consumed. These values were then compared with the entries made in the diet questionnaires. Similarly, for the food record entries of fish dishes, meat dishes, poultry, and so on (the items listed in the FFQ part of the diet questionnaire), a daily average consumption frequency was calculated. Based on these calculated consumption frequencies, each food group was recoded according to the seven-point scale of the FFQ part of the diet questionnaire, to allow the comparison between the two methods.

### Statistical analyses

Descriptive statistics are reported as percentages for categorical data, median (interquartile range) for non-normally distributed continuous data, and mean ± standard deviation (SD) for normally distributed continuous data. The respective statistical comparisons were performed using Chi squared test, Mann-Whitney U-test, and t-test.

Exploratory factor analysis (maximal likelihood and varimax rotation) was conducted to reveal underlying constructs within the FFQ in both the LPS-diet study and the validation study. In these analyses, the number of factors identified was based on eigenvalues >1.0, and food items with factor loading |≥0.20| with a particular factor, were included. The factor score was the sum of the scores for all items associated with that particular factor multiplied by its corresponding factor loading. In the validation study, the factor analysis was additionally conducted for the 6-day food record data. Within each dietary pattern, for which we could identify a reasonable match between the FFQ and the food record derived patterns, quartiles were formed based on the factor scores. The proportion of individuals classified into the same quartile, the same or adjacent quartile, or opposite quartile by the FFQ and the food record, was calculated.

The Spearman correlation coefficient was used to calculate the correlation between the factor scores of the matched dietary patterns in the validation study. Moreover, it was used to explore the correlations between serum LPS activity and dietary variables (both the factor scores of the FFQ-derived dietary patterns, and the energy, macronutrient, and fibre intake calculated from the food records). The independent associations between these dietary variables, and serum LPS activity were investigated in a multivariable generalized linear model.

Multivariable nutrient density substitution model was used to examine the associations between macronutrient intakes and LPS activity, as previously described^42^. In these analyses, LPS activity was entered as a dependent variable in the generalized linear regression analysis and, together with energy intake and selected confounders, dietary macronutrients (per 5 E%) were entered as independent variables. However one of the macronutrients, in each analysis, was left out. The results can be interpreted as an increase or decrease in LPS activity related to the isoenergetic (5 E%) substitution of a given macronutrient in the model with the macronutrient that was excluded from the model. As an example, in an equation: LPS activity = β_0_ + β_1_ (5 E% from carbohydrates) + β_2_ (5 E% from proteins) + β_3_ (5 E% from alcohol) + β_4_ (kcal), β_1_ can be interpreted as the change in the LPS activity when dietary carbohydrate intake is increased by 5 E% at the expense of fats, which was excluded from the model. Full models were corrected for sample storage time (as the LPS activity may reduce with increasing sample storage time), age, sex, smoking, physical activity, and diabetic nephropathy status. The substitution models with fatty acid intake were additionally adjusted for trans fatty acid intake.

A two-tailed *P* value < 0.05 was considered statistically significant. All data were analysed using IBM SPSS Statistics for Windows, Version 22.0 (IBM Corp, Armonk, NY, USA).

## Electronic supplementary material

Supplementary material
