# Supplementary material for: Dietary patterns reflecting healthy food choices are associated with lower serum LPS activity
Source: Sci Rep. 2017 Jul 26;7:6511. doi: 10.1038/s41598-017-06885-7 (PMC5529547; doi:10.1038/s41598-017-06885-7)
Supplement: Supplementary file 1 — Supplementary material [file 41598_2017_6885_MOESM1_ESM.pdf]

Dietary patterns reflecting healthy food choices are associated with lower serum LPS activity

Aila J Ahola, Mariann I Lassenius, Carol Forsblom, Valma Harjutsalo, Markku Lehto, and Per-Henrik Groop

Supplementary Table 1 Basic characteristics of the validation study population

	n = 1171
Men (%)	41
Age (years)	47 (36 – 55)
Diabetes duration (years)	27 (17 – 38)
Body mass index (kg/m^2)	25 (23 – 28)
Smoking (%)	14
Systolic blood pressure (mmHg)	135 (124 – 149)
Diastolic blood pressure (mmHg)	77 (71 – 84)
HbA_{1c} (mmol/mol)	64 (56 – 73)
HbA_{1c} (%)	8.0 (7.3 – 8.7)
Triglycerides (mmol/L)	0.90 (0.65 – 1.26)
Total cholesterol (mmol/L)	4.4 (3.9 – 5.0)
HDL cholesterol (mmol/L)	1.7 (1.4 – 2.0)

Data are presented as frequency or median (interquartile range)

Supplementary Table 2 Associations between reported consumption of food items in the diet questionnaire and in the 6-day food record

	Diet questionnaire	6-day food record		
	Mean ± SD or %	Mean ± SD or %	Correlation coefficient	*P*-value
Coffee (cups per day)	3.8 ± 2.8	3.0 ± 2.0	0.77	<0.001
Filtered coffee (%)	82.0	84.9		<0.001
Tea (cups per day)	0.8 ± 1.4	0.6 ± 1.0	0.72	<0.001
Milk (glasses per day)	1.4 ± 1.5	0.9 ± 1.0	0.82	<0.001
Fat free milk (%)	44.2	47.1		<0.001
Sour milk (glasses per day)	0.3 ± 0.7	0.2 ± 0.4	0.76	<0.001
Fat free sour milk (%)	24.0	20.3		<0.001
Yoghurt or curd (portions per day)	0.8 ± 0.7	0.5 ± 0.5	0.68	<0.001
Low fat yoghurt or curd (%)	53.4	41.8		<0.001
Bread (slices per day)	4.7 ± 2.1	4.6 ± 2.1	0.63	<0.001
Rye bread (%)	85.6	91.0		<0.001
Vegetable based spread (%)	67.3	64.4		<0.001

For continuous variables, data are presented as mean ± standard deviation (e.g. number of cups of coffee per day reported in the diet questionnaire and in the 6-day food record). For these data Pearson correlation coefficients are also provided. For categorical variables, percentages are shown (e.g. percentage of individuals reporting consumption of filtered coffee in the diet questionnaire and in the 6-day food record).

Supplementary Table 3 Frequencies of monthly intake of the food groups according to the food frequency questionnaire (FFQ) and the 6-day food record

	FFQ	6-day food record		
	Mean ± SD	Mean ± SD	Correlation coefficient	*P*-value
Fish dishes	5.7 ± 5.1	8.3 ± 7.9	0.42	<0.001
Meat dishes	11.7 ± 9.3	17.5 ± 9.9	0.43	<0.001
Poultry	8.5 ± 7.3	6.5 ± 6.9	0.40	<0.001
Sausages and cold cuts	19.2 ± 17.8	27.0 ± 18.4	0.58	<0.001
Eggs	4.3 ± 5.9	4.6 ± 6.5	0.39	<0.001
Legumes	5.0 ± 7.3	4.6 ± 7.5	0.23	<0.001
Fresh vegetables	31.6 ± 18.2	34.5 ± 17.9	0.57	<0.001
Cooked vegetables	13.2 ± 12.0	14.3 ± 10.5	0.46	<0.001
Potatoes	17.1 ± 11.8	19.9 ± 11.8	0.55	<0.001
Pasta and rice	8.8 ± 7.8	9.6 ± 8.9	0.42	<0.001
Fruits and berries	28.1 ± 18.7	37.2 ± 19.3	0.53	<0.001
Cheese, ≥20% fat	8.6 ± 14.2	17.3 ± 18.1	0.51	<0.001
Cheese, <20% fat	21.0 ± 19.7	20.5 ± 20.1	0.52	<0.001
Yoghurt and curd	19.4 ± 16.7	18.6 ± 14.8	0.71	<0.001
Ice cream	2.5 ± 4.3	3.9 ± 5.8	0.31	<0.001
Soft drinks	8.7 ± 15.2	6.2 ± 12.2	0.55	<0.001
Sweet pastries	7.6 ± 9.8	9.2 ± 12.6	0.25	<0.001
Sweets and chocolate	6.7 ± 8.7	9.2 ± 2.6	0.48	<0.001

Supplementary Table 4 Factor loadings of the dietary patterns formed from the food frequency questionnaire (FFQ) and 6-day food record

	**Healthy**		**Traditional**		**Sweets**		**Healthy snack**		**Modern**		**Cheese**	
	FFQ	6DFR	FFQ	6DFR	FFQ	6DFR	FFQ	6DFR	FFQ	6DFR	FFQ	6DFR
Fish dishes	0.27	0.24										
Meat dishes			0.89	0.78					0.33			
Poultry									0.63	0.22		
Sausages and cold cuts			0.22	0.23								
Eggs											0.24	
Legumes	0.59	0.33										
Fresh vegetables	0.39	0.30					0.34	0.39				
Cooked vegetables	0.69	0.81							0.21			
Potatoes			0.42	0.34								
Pasta and rice									0.44	0.98		
Fruits and berries	0.27						0.60	0.80				
High-fat cheese											0.58	-0.31
Low-fat cheese							0.28	0.27			-0.43	0.88
Yoghurt and curd							0.51	0.26				
Ice cream					0.24	0.27						
Soft drinks												
Sweet pastries					0.72	0.96						
Sweets and chocolate					0.50	0.97						

A high positive loading of a food item indicates its strong positive association with a dietary pattern, while a negative loading indicates that the food item is inversely associated with a given pattern.

Supplementary Table 5 The proportion of individuals classified into the same, same or adjacent, and opposite quartiles, formed from the factor scores of the factor analysis of the food frequency questionnaire and the 6-day food record data, and the correlation coefficients of the factor scores for the dietary patterns

	Classified into				
	same quartile	same or adjacent quartiles	opposite quartiles	Correlation coefficient	*P*-value
Chance	25.0	62.5	12.5		
Healthy snack	40.0	81.0	4.9	0.46	<0.001
Traditional	34.8	76.0	4.5	0.41	<0.001
Sweets	33.3	74.0	5.4	0.33	<0.001
Snack	40.7	80.2	3.1	0.51	<0.001
Modern	32.9	71.5	8.0	0.23	<0.001
Cheese	41.9	78.7	4.8	-0.44	<0.001

Supplementary Table 6 The diet questionnaire

Question	Type of data	Included in the validation study
How many cups of coffee do you usually drink per day?	Numerical	Yes
How is the coffee made, that you usually drink?	*Multiple choice: choose the one that best describes your habits*<br>Filtered coffee, or coffee made with coffee maker<br>Coffee cooked in a pot, or coffee made with espresso machine<br>Instant coffee<br>Other type of coffee, specify<br>I do not usually drink coffee	Yes
How many cups of tea do you usually drink per day?	Numerical	Yes
How many glasses (2 dl) of milk do you usually drink per day?	Numerical	Yes
What kind of milk do you usually drink?	*Multiple choice: choose the one that best describes your habits*<br>Skim milk or lactose free skim milk drink<br>Milk or lactose free milk drink with 1 – 1,5 % fat<br>Full fat milk or unprocessed milk from farm<br>Organic milk with 1 – 1,5 % fat<br>Skim organic milk<br>Milk with stanols or sterols added<br>Other type of milk, specify<br>I do not usually drink milk	Yes
How many glasses (2 dl) of sour milk do you usually drink per day?	Numerical	Yes
What kind of sour milk do you usually drink?	*Multiple choice: choose the one that best describes your habits*<br>Skim sour milk<br>Sour milk with 0,5 – 1,5 % fat<br>Sour milk with 2 % or more fat<br>Skim organic sour milk	Yes

	Sour milk with stanols<br>Other type of sour milk, specify<br>I do not usually drink sour milk	
How many portions (2dl) of yoghurt/curd do you usually eat per day?	Numerical	Yes
What kind of yoghurt/curd do you usually eat?	*Multiple choice: choose the one that best describes your habits*<br>Skim or low fat yoghurt/curd<br>Yoghurt/curd with regular amount of fat<br>I do not usually eat yoghurt/curd	Yes
How many slices of bread do you usually eat per day?	Numerical	Yes
What kind of bread do you usually eat almost daily?	*Multiple choice: choose all that apply*<br>Rye bread or bread made mainly of rye<br>Bread made from mixed flour<br>Wheat bread<br>Rye crisp<br>Other type of bread, specify__________________<br>I do not usually eat bread	Yes
What kind of spread do you usually consume on bread?	*Multiple choice: choose the one that best describes your habits*<br>I do not use any spread on bread<br>Butter<br>Butter and vegetable oil mix<br>Margarine with 70 – 80 % fat<br>Low-fat spread with 65 % fat or less<br>Margarine with stanols or sterols<br>Unripened cheese, cheese spread or like<br>I do not usually eat bread	Yes
What kind of fat do you usually use for cooking at home?	*Multiple choice: choose the one that best describes your habits*<br>I do not use any fat for cooking<br>Vegetable oil (for example olive oil, canola oil)	No. Cooking fats were seldom reported in the food record.

	Butter<br>Butter and vegetable oil mix<br>Margarine or fluid margarine<br>Cooking margarine<br>Margarine with stanols or sterols<br>I do not cook at home	
How often do you add salt to your food at table?	*Multiple choice: choose the one that best describes your habits*<br>Almost never<br>Usually when food does not taste salty enough<br>Almost always prior tasting	No. The timing of the salt addition was not specified in the food record.
Do you prefer low-salt foodstuffs?	*Multiple choice: choose the one that best describes your habits*<br>Never<br>Seldom<br>Sometimes<br>Mostly<br>Always	No. Participants rarely reported the salt content of the commercial products.
Have you consumed foods or supplements with lactic acid bacteria over the preceding month?	No / Yes, trade name and dose	No. Supplements were not recorded in the food record.
Have you used any dietary supplements (for example vitamin or mineral supplements or oil capsules) over the preceding month?	No / Yes, trade name and dose	No. Supplements were not recorded in the food record.
Have you used any natural products (i.e. herbal remedies or homeopathic products) over the preceding month?	No / Yes, trade name and dose	No. Supplements were not recorded in the food record.
Have you received any instructions concerning your diet from someone working at the health care system?	*Multiple choice: choose all that apply*<br>No<br>Yes, from the dietitian<br>Yes, from the diabetes nurse<br>Yes, from the doctor<br>Yes, from someone else. From whom?	No. Dietary recommendations were not queried in the food record.

When have you received instructions regarding your diet from the health care system?	*Multiple choice: choose all that apply*<br>I have not received any instructions<br>At the time of diabetes diagnosis<br>At the time of another diagnosis: when, which diagnosis?<br>At another time: when?	No. Dietary recommendations were not queried in the food record.
Do you follow the dietary instructions given to you at the health care system?	*Multiple choice: choose the one that best describes your habits*<br>I have not received any instructions<br>Never<br>Seldom<br>Sometimes<br>Most of the time<br>Always	No. Dietary recommendations were not queried in the food record.
Do you follow any special diet at the moment?	*Multiple choice: choose all that apply*<br>No<br>Low-lactose or lactose-free diet based on the diagnosis or recommendation given at health care system<br>Low-lactose or lactose-free diet on my own decision<br>Gluten-free diet based on the diagnosis or recommendation given at health care system<br>Gluten-free diet on my own decision<br>Protein restricted diet based on the diagnosis or recommendation given at health care system<br>Protein restricted diet on my own decision<br>Vegetarian or vegan diet based on the diagnosis or recommendation given at health care system<br>Vegetarian or vegan diet on my own decision<br>Vegetarian or vegan diet based on other reasons<br>Other special diet, specify	No. Special diets were not specifically reported in the food record.
How often have you consumed the following food items over the preceding month?	*Multiple choice: choose the one that best describes your habits*	

	Several times a day	
	Once a day	
	4-6 times a week	
	2-3 times a week	
	Once a week	
	1-3 times a month	
	Less frequently or never	
Fish dishes		Yes
Meat dishes (cattle, pork, lamb, game)		Yes
Poultry (chicken, broiler, or other bird)		Yes
Sausages and cold cuts		Yes
Eggs (boiled, fried, or as omelets)		Yes
Peas, beans, and other legumes		Yes
Fresh vegetables (salads and such)		Yes
Cooked vegetables (excluding potatoes)		Yes
Potatoes		Yes
Pasta and rice		Yes
Fruits and berries (excluding juices)		Yes
Cheese containing 20% fat or more		Yes
Low-fat cheese (less than 20% fat)		Yes
Yoghurt, curd		Yes
Ice cream		Yes
Soft drinks		Yes
Sweet pastries (cakes, cookies and such)		Yes
Sweets and chocolate		Yes
Fried and grilled foods		No. The cooking methods were not always evident in the food records.

Supplementary information

The Finnish Diabetic Nephropathy Study Centers

Anjalankoski Health Center	S.Koivula, T.Uggeldahl
Central Finland Central Hospital, Jyväskylä	T.Forslund, A.Halonen, A.Koistinen, P.Koskiaho, M.Laukkanen, J.Saltevo, M.Tiihonen
Central Hospital of Åland Islands, Mariehamn	M.Forsen, H.Granlund, A.-C.Jonsson, B.Nyroos
Central Hospital of Kanta-Häme, Hämeenlinna	P.Kinnunen, A.Orvola, T.Salonen, A.Vähänen
Central Hospital of Kymenlaakso, Kotka	R.Paldanius, M.Riihelä, L.Ryysy
Central Hospital of Länsi-Pohja, Kemi	H.Laukkanen, P.Nyländen, A.Sademies
Central Ostrobothnian Hospital District, Kokkola	S.Anderson, B.Asplund, U.Byskata, P.Liedes, M.Kuusela, T.Virkkala
City of Espoo Health Center:	
Espoonlahti	A.Nikkola, E.Ritola
Tapiola	M.Niska, H.Saarinen
Samaria	E.Oukko-Ruponen, T.Virtanen
Viherlaakso	A.Lyytinen
City of Helsinki Health Center:	
Puistola	H.Kari, T.Simonen
Suutarila	A.Kaprio, J.Kärkkäinen, B.Rantaeskola
Töölö	P.Kääriäinen, J.Haaga, A-L.Pietiläinen
City of Hyvinkää Health Center	S.Klemetti, T.Nyandoto, E.Rontu, S.Satuli-Autere
City of Vantaa Health Center:	
Korso	R.Toivonen, H.Virtanen
Länsimäki	R.Ahonen, M.Ivaska-Suomela, A.Jauhiainen
Martinlaakso	M.Laine, T.Pellonpää, R.Puranen
Myyrmäki	A.Airas, J.Laakso, K.Rautavaara
Rekola	M.Erola, E.Jatkola
Tikkurila	R.Lönnblad, A.Malm, J.Mäkelä, E.Rautamo
Heinola Health Center	P.Hentunen, J.Lagerstam
Helsinki University Central Hospital, Department of Medicine, Division of Nephrology	M.Feodoroff, D.Gordin, O.Heikkilä, K.Hietala, J.Fagerudd, M.Korolainen, L.Kyllönen, J.Kytö, S.Lindh, K.Pettersson-Fernholm, M.Rosengård-Bärlund, A.Sandelin, L.Thorn, J.Tuomikangas, T.Vesisenaho, J.Wadén
Herttoniemi Hospital, Helsinki	V.Sipilä
Hospital of Lounais-Häme, Forssa	T.Kalliomäki, J.Koskelainen, R.Nikkanen, N.Savolainen, H.Sulonen, E.Valtonen
Hyvinkää Hospital	L. Norvio, A.Hämäläinen
Iisalmi Hospital	E.Toivanen
Jokilaakso Hospital, Jämsä	A.Parta, I.Pirttiniemi
Jorvi Hospital, Helsinki University Central Hospital	S.Aranko, S.Ervasti, R.Kauppinen-Mäkelin, A.Kuusisto, T.Leppälä, K.Nikkilä, L.Pekkonen
Jyväskylä Health Center, Kyllö	K.Nuorva, M.Tiihonen
Kainuu Central Hospital, Kajaani	S.Jokelainen, K.Kananen, M.Karjalainen, P.Kemppainen, A-M.Mankinen, A.Reponen M.Sankari
Kerava Health Center	H.Stuckey, P.Suominen
Kirkkonummi Health Center	A.Lappalainen, M.Liimatainen, J.Santaholma
Kivelä Hospital, Helsinki	A.Aimolahti, E.Huovinen
Koskela Hospital, Helsinki	V.Ilkka, M.Lehtimäki
Kotka Health Center	E.Pälikkö-Kontinen, A.Vanhanen
Kouvola Health Center	E.Koskinen, T.Siitonen
Kuopio University Hospital	E.Huttunen, R.Ikäheimo, P.Karhapää, P.Kekäläinen, M.Laakso, T.Lakka, E.Lampainen, L.Moilanen, S. Tanskanen L.Niskanen, U.Tuovinen, I.Vauhkonen, E.Voutilainen
Kuusamo Health Center	T.Kääriäinen, E.Isopoussu
Kuusankoski Hospital	E.Kilkki, I.Koskinen, L.Riihelä
Laakso Hospital, Helsinki	T.Meriläinen, P.Poukka, R.Savolainen, N.Uhlenius
Lahti City Hospital	A.Mäkelä, M.Tanner
Lapland Central Hospital, Rovaniemi	L.Hyvärinen, K.Lampela, S.Pöykkö, T.Rompasaari, S.Severinkangas, T.Tulokas
Lappeenranta Health Center	P. Erola, L.Härkönen, P.Linkola, T.Pekkanen, I.Pulli, E.Repo

Lohja Hospital	T.Granlund, K.Hietanen, M.Porrassalmi, M.Saari, T.Salonen,
M.Tiikkainen,	
Länsi-Uusimaa Hospital, Tammisaari	I.-M.Jousmaa, J.Rinne
Loimaa Health Center	A.Mäkelä, P.Eloranta
Malmi Hospital, Helsinki	H.Lanki, S.Moilanen, M.Tilly-Kiesi
Mikkeli Central Hospital	A.Gynther, R.Manninen, P.Nironen, M.Salminen, T.Vänttinen
Mänttä Regional Hospital	I.Pirttiniemi, A-M.Hänninen
North Karelian Hospital, Joensuu	U-M.Henttula, P.Kekäläinen, M.Pietarinen, A.Rissanen, M.Voutilainen
Nurmijärvi Health Center	A.Burgos, K.Urtamo
Oulaskangas Hospital, Oulainen	E.Jokelainen, P-L.Jylkkä, E.Kaarlela, J.Vuolaspuro
Oulu Health Center	L.Hiltunen, R.Häkkinen, S.Keinänen-Kiukaanniemi
Oulu University Hospital	R.Ikäheimo
Päijät-Häme Central Hospital	H.Haapamäki, A.Helanterä, S.Hämäläinen, V.Ilvesmäki, H.Miettinen
Palokka Health Center	P.Sopanen, L.Welling
Pieksämäki Hospital	V.Sevtsenko, M.Tamminen
Pietarsaari Hospital	M-L.Holmbäck, B.Isomaa, L.Sarelin
Pori City Hospital	P.Ahonen, P.Merisalo, E.Muurinen, K.Sävelä
Porvoo Hospital	M.Kallio, B.Rask, S.Rämö
Raahe Hospital	A.Holma, M.Honkala, A.Tuomivaara, R.Vainionpää
Rauma Hospital	K.Laine, K.Saarinen, T.Salminen
Riihimäki Hospital	P.Aalto, E.Immonen, L.Juurinen
Salo Hospital	A.Alanko, J.Lapinleimu, P.Rautio, M.Virtanen
Satakunta Central Hospital, Pori	M.Asola, M.Juhola, P.Kunelius, M.-L.Lahdenmäki, P.Pääkkönen, M.Rautavirta
Savonlinna Central Hospital	T.Pulli, P.Sallinen, M.Taskinen, E.Tolvanen, T.Tuominen H.Valtonen, A.Vartia, S-L.Viitanen
Seinäjoki Central Hospital	O.Antila, E.Korpi-Hyövälti, T.Latvala, E.Leijala, T.Leikkari, M.Punkari N.Rantamäki, H.Vähävuori
South Karelia Central Hospital, Lappeenranta	T.Ensala, E.Hussi, R.Härkönen, U.Nyholm, J.Toivanen
Tampere Health Center	A.Vaden, P.Alarotu, E.Kujansuu, H.Kirkkopelto-Jokinen, M.Helin, S.Gummerus, L.Calonius, T.Niskanen, T.Kaitala, T.Vatanen
Tampere University Hospital	P. Hannula, I.Ala-Houhala, R.Kannisto, T.Kuningas, P.Lampinen,
M.Määttä,	
	H.Oksala, T.Oksanen, A.Putila, H.Saha, K.Salonen, H.Tauriainen, S.Tulokas
Tiirismaa Health Center, Hollola	T.Kivelä, L.Petlin, L.Savolainen
Turku Health Center	A.Artukka, I.Hämäläinen, L.Lehtinen, E.Pyysalo, H.Virtamo,
M.Viinikkala, M.Vähätalo	
Turku University Central Hospital	K.Breitholz, R.Eskola, K.Metsärinne, U.Pietilä, P.Saarinen, R.Tuominen, S.Äyräpää
Vaajakoski Health Center	K.Mäkinen, P.Sopanen
Valkeakoski Regional Hospital	S.Ojanen, E.Valtonen, H.Ylönen, M.Rautiainen, T.Immonen
Vammala Regional Hospital	I.Isomäki, R.Kroneld, L.Mustaniemi, M.Tapiolinna-Mäkelä
Vasa Central Hospital	S.Bergkulla, U.Hautamäki, V-A.Myllyniemi, I.Rusk
